# Pharmacokinetic considerations in testing hypoxic cell radiosensitizers in mouse tumours.

**DOI:** 10.1038/bjc.1979.55

**Published:** 1979-03

**Authors:** J. M. Brown, N. Y. Yu, P. Workman

## Abstract

Bilateral kidney ligation of mice immediately before injection of misonidazole (MIS) prolongs the plasma half-life of this radiosensitizer from about 2 h (in normal mice) to 10-11 h, similar to that in man. Kidney ligation does not, however, change the relative proportions of MIS and its O-demethylated metabolite, Ro-05-9963, for the first 12 h after MIS injection. Kidney ligation was used with the two radiosensitizers, MIS and Ro-05-9963, to investigate the influence of plasma half-life both on peak plasma levels and on the tumour/plasma ratio of sensitizer concentration in the EMT6 mouse tumour. Although the acute LD50 of Ro-05-9963 in normal mice was twice that of MIS, this apparent advantage was offset by peak tumour levels 50% or less of those achieved by equimolar injected doses of MIS. However, by comparing the plasma and tumour levels in mice in which the drug half-lives were prolonged by bilateral kidney ligation, it was concluded that the lower plasma and tumour levels of Ro-05-9963 were a result of its shorter plasma half-life, rather than of an intrinsic barrier to tumour penetration. Because of this rapid clearance, the radiosensitization produced by Ro-05-9963 was less than that produced by equimolar injected doses of MIS. As this difference did not occur in kidney-ligated mice, and hence would not be expected to occur in man, the comparison of MIS and Ro-05-9963 in mice produces an artificially low radiosensitization for Ro-05-9963 and possibly also for other compounds with short plasma half-lives. Although the short plasma half-life of Ro-05-9963 appeared to be responsible for its low peak plasma concentration, it did not produce a low tumour/plasma ratio. Within the limits of plasma nitroimidazole half-lives investigated (0.5-10 h) the tumour/plasma ratio was insensitive to plasma half-life, being 50-70% for both MIS and Ro-05-9963 in both normal and kidney-ligated mice. It is concluded that the common assumption that tumour/plasma ratios of MIS in the mouse are less than those in man is unjustified.


*Br. J. Cancer* (1979) **39**, 310

### PHARMACOKINETIC CONSIDERATIONS IN TESTING HYPOXIC CELL RADIOSENSITIZERS IN MOUSE TUMOURS

J. M. BROWN,† N. Y. YU† AND P. WORKMAN*

*From the †Department of Radiology, Stanford University School of Medicine, Stanford, California 94305 and *MRC Clinical Oncology and Radiotherapeutics Unit, Hills Road, Cambridge*

Received 10 July 1978 Accepted 17 November 1978

**Summary.—Bilateral kidney ligation of mice immediately before injection of misonidazole (MIS) prolongs the plasma half-life of this radiosensitizer from about 2 h (in normal mice) to 10–11 h, similar to that in man. Kidney ligation does not, however, change the relative proportions of MIS and its O-demethylated metabolite, Ro-05-9963, for the first 12 h after MIS injection. Kidney ligation was used with the two radiosensitizers, MIS and Ro-05-9963, to investigate the influence of plasma half-life both on peak plasma levels and on the tumour/plasma ratio of sensitizer concentration in the EMT6 mouse tumour.**

**Although the acute $LD_{50}$ of Ro-05-9963 in normal mice was twice that of MIS. this apparent advantage was offset by peak tumour levels 50% or less of those achieved by equimolar injected doses of MIS. However, by comparing the plasma and tumour levels in mice in which the drug half-lives were prolonged by bilateral kidney ligation, it was concluded that the lower plasma and tumour levels of Ro-05-9963 were a result of its shorter plasma half-life, rather than of an intrinsic barrier to tumour penetration. Because of this rapid clearance, the radiosensitization produced by Ro-05-9963 was less than that produced by equimolar injected doses of MIS. As this difference did not occur in kidney-ligated mice, and hence would not be expected to occur in man, the comparison of MIS and Ro-05-9963 in mice produces an artificially low radiosensitization for Ro-05-9963 and possibly also for other compounds with short plasma half-lives.**

**Although the short plasma half-life of Ro-05-9963 appeared to be responsible for its low peak plasma concentration, it did not produce a low tumour/plasma ratio. Within the limits of plasma nitroimidazole half-lives investigated (0·5–10 h) the tumour/plasma ratio was insensitive to plasma half-life, being 50–70% for both MIS and Ro-05-9963 in both normal and kidney-ligated mice. It is concluded that the common assumption that tumour/plasma ratios of MIS in the mouse are less than those in man is unjustified.**

THE HYPOXIC CELL RADIOSENSITIZER misonidazole (1-(2-nitroimidazol-l-yl)-3-methoxypropan-2-ol, Ro-07-0582, MIS) has been shown to be highly effective in sensitizing hypoxic tumour cells (both *in vitro* and *in vivo*) to cell killing by radiation (Asquith *et al.*, 1974; Denekamp *et al.*, 1974; Brown, 1975). There is also evidence of radiosensitization of human tumours by this drug (Thomlinson *et al.*, 1976). However, it is also apparent that the neurological side effects of MIS limit tumour levels of the drug to below optimum values for radiosensitization (Dische *et al.*, 1977; Urtasun *et al.*, 1978). Indeed, if MIS were to be given in a conventional fractionation regime, the development of peripheral neuropathy would limit plasma concentrations to ~20 μg/ml, for which sensitization-enhancement ratios of the hypoxic cells would not be expected to be greater than 1·1 to 1·3.

Although it is possible that such a small degree of radiosensitization of the hypoxic tumour cells could improve local control for some tumours (owing to their steep dose-response curves), it is clear that MIS is not the "optimum" hypoxic cell radiosensitizer for clinical application. Such a radiosensitizer would be as effective as MIS in its radiosensitizing ability, but only 1/10th as toxic. Several groups are now pursuing such a goal (Adams, 1977; Brown *et al.*, 1978; Wardman, 1977).

It became apparent early in our studies that the O-demethylated metabolite of MIS (1-(2-nitroimidazol-1-yl)-2,3-propandiol, Ro-05-9963) was less toxic in mice, and hence might be a better drug for clinical application. However, we also found that Ro-05-9963 did not give as high a level of radiosensitization of tumours *in vivo* as MIS injected at equimolar doses (Brown *et al.*, 1978), though this could be entirely explained by lower tumour concentrations (Brown *et al.*, in preparation). In order to determine whether the lower tumour concentrations of Ro-05-9963 than MIS are due to the short plasma half-life of Ro-05-9963 in the mouse, we have examined the influence of plasma half-life on tumour concentration. Specifically the two questions were:

(a) How much does the plasma half-life affect the peak plasma level?
(b) How much does the plasma half-life affect the tumour/plasma ratio of drug concentrations?

### MATERIALS AND METHODS

A description of the procedures for implantation of tumours, and irradiation and assay of the viability of the tumour cells has been published previously (Brown, 1975). Briefly, the EMT6/St/lu tumour was transplanted into 3-month-old, syngeneic BALB/c female mice, weighing 20–25 g, and irradiated at a diameter of 6–7 mm. Immediately after irradiation the tumours were removed and minced. The only modification was that the single-cell suspensions were prepared by a 30 min disaggregation using an enzyme "cocktail" of 0·05% pronase, 0·02% DNAase and 0·02% collagenase (Brown *et al.*, in preparation). Cell viability was assayed by plating appropriate dilutions of the tumour cells into Waymouth's medium containing 15% foetal calf serum in polystyrene Petri dishes, and counting colonies 13 days later.

Both MIS (mol.wt 201) and Ro-05-9963 (mol.wt 187) were dissolved in physiological saline or Hanks' medium immediately before use, at a concentration sufficient to inject the desired dose i.p. in 0·04–0·05 ml/g body wt.

Bilateral kidney ligation was performed on anaesthetized mice by exteriorizing the kidneys through a 1 cm incision in the skin and abdominal wall and tying a ligature around the renal artery and vein at the renal pelvis. The abdominal incision, which was adjoining and parallel to the line of attachment of the abdominal wall with the dorsal muscles, was then closed tightly with 4 or 5 stitches, and the skin incision closed with metal clips. The sham operation was identical in all respects except for ligation of the renal artery and vein. Mice were injected with MIS or Ro-05-9963 when they had fully recovered from the anaesthetic ($\sim$2 h after the operation).

Plasma (or whole blood) and tumour levels of 2-nitroimidazoles were determined in different ways in the two laboratories represented.

In the first method, represented in Tables I and II and Fig. 1, tumour and plasma levels were determined on killing of individual mice, and were measured spectrophotometrically using UV absorption at 318 nm. This measures the total concentration of all 2-nitroimidazoles in the sample. The procedure used was a modification of that described by Chapman *et al.* (1974). To measure plasma levels, 0·4 ml of plasma (collected from heparinized whole blood by cardiac puncture at the time of killing) was added to 2·6 ml absolute ethanol. The mixture was vortexed for 1 min, allowed to stand for 10 min and then centrifuged for 5 min at high speed (1000 *g*). Tumour levels were determined by preparing a 20% (w/v) homogenate of tumour in distilled water, and adding a 0·4 ml aliquot of homogenate to 2·6 ml absolute ethanol as described above. The recovery of drug added to plasma or tumour samples was 90–100%.

In experiments to measure the half-life and principal metabolites of MIS in control, sham-operated and kidney-ligated mice, the concentrations of 2-nitroimidazoles were determined by the second method: reversed-phase high-performance liquid chromotography (HPLC). At appropriate times after MIS administration, serial blood samples were taken from the tail of the same mouse. Duplicate 5 $\mu$l samples were collected in Microcap pipettes (Drummond Scientific Company), mixed with 45 $\mu$l water and stored at −20°C. Finally (usually at 10–12·5 h) the mice were bled by cardiac puncture and the undiluted heparinized whole blood was stored at −20°C. Blood samples were analysed for MIS and Ro-05-9963 as described previously for plasma (Workman *et al.*, 1978*a*). Concentrations in whole blood were identical to those in plasma.

Chromatograms of blood samples from kidney-ligated animals exhibited larger peaks at the solvent front than those from control and sham-operated mice. MIS is excreted in mouse urine partly as glucuronidase-hydrolysable conjugate(s) of the parent drug (Flockhart *et al.*, 1978*c*). We therefore reasoned that some of the material eluting at the solvent front might include such polar conjugates. To test this, samples of 10–12·5 h whole blood were incubated in the dark at 37°C with an equal volume of Glucurase (Sigma Chemical Company). (Glucurase is a solution of bovine liver $\beta$-glucuronidase buffered at pH 5; activity=5000 Sigma units/ml.) Aliquots were removed at 24 h and 48 h and analysed by HPLC. This treatment caused a decrease in the peak at the solvent front and a concomitant increase in the amount of MIS and Ro-05-9963. These changes were complete by 24 h. The treatment did not alter the amount of MIS and Ro-05-9963 in whole blood from control and sham-operated mice. The difference in concentration between digested and undigested whole blood gave the amount of glucuronidase-hydrolysable conjugates of MIS and Ro-05-9963. In accordance with previous convention (Flockhart *et al.*, 1978*c*), we shall refer to these as the O-glucuronide conjugates of MIS and Ro-05-9963.

### RESULTS

### *Acute toxicity of MIS and Ro*-05-9963

The values obtained (at Stanford, in the same experiment on the same batch of mice) for the $LD_{50/2d}$ (and 95% confidence intervals) of MIS and Ro-05-9963 were as follows:

(i) MIS: 9·1 (6·5–12·8) mmol/kg or 1833 (1305–2576) mg/kg.
(ii) Ro-05-9963: 16·6 (11·8–23·3) mmol/kg or 3104 (2212–4355) mg/kg.

The lower toxicity of Ro-05-9963 (by a factor of 1·7–2·2) has been found by others (Flockhart *et al.*, 1978*b*; Stone, personal communication, 1978). For both drugs the deaths of the animals were rapid: even at doses close to the $LD_{50}$ only an occasional mouse died beyond 5–6 h after injection. This, and the comatose nature of the mice, suggest death by a neurological mechanism.

### *Drug levels in plasma and tumour*

Earlier radiation experiments with EMT6 tumours showed that low doses of Ro-05-9963 produced radiosensitization of the tumours *in vivo*, but unlike similar doses of MIS, the drug was not dose-modifying (Brown *et al.*, 1978). This suggested that the drug was not penetrating adequately to all the hypoxic tumour cells. Further experiments have been performed with 3 tumours (the EMT6 tumour, the KHT sarcoma and the RIF-1 sarcoma) and it has been found that although at injected doses of 935 mg/kg (5 mmol/kg) Ro-05-9963 is dose-modifying, it never achieved as high a degree of radiosensitization as MIS at equimolar injected doses (Brown *et al.*, in preparation).

Since Ro-05-9963 and MIS have equal electron affinities, they would be expected to be equally effective as radiation sensitizers and, in fact, this appears to be the case *in vitro*. Adams *et al.* (1976) reported that Ro-05-9963 was slightly less effective than MIS in radiosensitizing V-79 cells, whereas Flockhart *et al.* (1968*b*) found that Ro-05-9963 was slightly more so. Thus it appeared that the most likely explanation of the lower radiosensitization by Ro-05-9963 *in vivo* would be the lower tumour concentration. Since we found maximum tumour radiosensitization at

TABLE I.—*Plasma and tumour concentrations* (mM)* *of misonidazole and Ro*-05-9963 30–45 *min after injection*

| Drug and dose | Time after inj. (min) | Plasma concn. | Tumour concn. | Tumour/ Plasma | 30–45 min means ± s.e. Tumour | 30–45 min means ± s.e. Tumour/ Plasma |
|---|---|---|---|---|---|---|
| MIS | 30 | 5·3 | 2·5 | 0·48 | | |
| 1000 mg/kg | 30 | 4·6 | 3·0 | 0·65 | | |
| (5 mmol/kg) | 30 | 5·6 | 2·6 | 0·47 | | |
| | 30 | 5·7 | 3·3 | 0·59 | | |
| | Mean ± s.e. | 5·3 ± 0·3 | 2·9 ± 0·2 | 0·55 ± 0·04 | | |
| | 37·5 | 4·5 | 3·1 | 0·69 | | |
| | 37·5 | 2·8 | 1·8 | 0·66 | | |
| | 37·5 | 4·6 | 3·0 | 0·66 | 2·6 ± 0·2 | 0·59 ± 0·02 |
| | 37·5 | 5·1 | 2·4 | 0·47 | | |
| | Mean ± s.e. | 4·2 ± 0·5 | 2·6 ± 0·3 | 0·62 ± 0·05 | | |
| | 45 | 4·3 | 2·7 | 0·63 | | |
| | 45 | 4·5 | 2·8 | 0·63 | | |
| | 45 | 4·2 | 2·7 | 0·63 | | |
| | 45 | 2·8 | 1·5 | 0·55 | | |
| | Mean ± s.e. | 4·0 ± 0·4 | 2·4 ± 0·3 | 0·61 ± 0·02 | | |
| Ro-05-9963 | 30 | 1·4 | 0·77 | 0·55 | | |
| 935 mg/kg | 30 | 2·8 | 1·5 | 0·57 | | |
| (5 mmol/kg) | 30 | 2·4 | 1·1 | 0·46 | | |
| | 30 | 2·6 | 1·4 | 0·53 | | |
| | Mean ± s.e. | 2·3 ± 0·3 | 1·2 ± 0·2 | 0·53 ± 0·02 | | |
| | 37·5 | 0·88 | 0·34 | 0·39 | | |
| | 37·5 | 2·4 | 2·4 | 1·0 | | |
| | 37·5 | 2·5 | 1·7 | 0·66 | 1·1 ± 0·2 | 0·60 ± 0·05 |
| | 37·5 | 2·4 | 1·7 | 0·72 | | |
| | Mean ± s.e. | 2·0 ± 0·4 | 1·5 ± 0·4 | 0·69 ± 0·12 | | |
| | 45 | 1·5 | 0·74 | 0·49 | | |
| | 45 | 1·5 | 0·76 | 0·51 | | |
| | 45 | 0·38 | 0·25 | 0·66 | | |
| | 45 | 1·2 | 0·75 | 0·62 | | |
| | Mean ± s.e. | 1·2 ± 0·3 | 0·63 ± 0·13 | 0·57 ± 0·04 | | |

* Determined by spectrophotometric assay.

30 min after injection, and our longest exposure in radiation experiments was 15 min, we compared the plasma and tumour concentrations of Ro-05-9963 and MIS from 30 to 45 min after an i.p. injection of equimolar injected doses in equal volumes of physiological saline. The results (Table I) show that, despite the inter-animal variation, the mean tumour concentration of Ro-05-9963 was less than 50% of that of MIS over the period studied. It can also be seen that this was due entirely to lower plasma levels of Ro-05-9963, since the tumour/plasma ratio of concentrations was the same for each drug.

At least two possibilities exist for the lower tumour concentrations of Ro-05-9963. Firstly, Ro-05-9963 is considerably less lipophilic than MIS (Adams *et al.*, 1976; Brown *et al.*, 1978) and hence may not be as able to penetrate the many lipoid barriers between the injection site and the nuclei of the tumour cells. Alternatively, a more rapid plasma clearance of Ro-05-9963 (which may itself be related to the lower lipophilicity) could have prevented the maximum uptake and retention in the tissues. The importance of deciding between these alternative hypotheses is that if inadequate absorption and distribution due to the lower lipophilicity were responsible for the lower tumour concentrations of Ro-05-9963, this property of the drug would be likely to apply both in man and mouse. However, if the lower tumour concentrations were due to the rapid clearance of Ro-05-9963

in the mouse, this might not be a problem in man, where the plasma half-life of MIS is considerably longer than in the mouse and might also be true for Ro-05-9963.

FIG. 1.—The plasma concentration of MIS (○) after an injection of 5 mmol/kg and of Ro-05-9963 (●) after an injection of 10 mmol/kg, as a function of time after i.p. injection into (Stanford) female BALB/c mice (±s.e.). The data were obtained by the UV spectrophotometric method on blood obtained on killing 4 mice at each interval. The half-lives and 95% confidence intervals were calculated by least-squares regression analysis.

Fig. 1 shows plasma levels as a function of time after injection in the same experiment, of doses of MIS and Ro-05-9963 designed to give the same initial plasma levels in BALB/c mice. (The importance of comparing plasma clearance half-lives of two drugs at the same peak level stems from the observation by ourselves and others (Brown, unpublished; Workman *et al.*, in preparation; Chin *et al.*, 1978): that the half-life is significantly longer with higher initial concentrations. This might be due to hypothermia and reduced metabolic rate at high drug doses, or it might be a corollary of an elimination rate with saturable kinetics.) It can be seen that despite equal peak plasma levels the plasma half-life was less for Ro-05-9963 (33 min) than for MIS (94 min). These data also confirm the conclusion from the previous experiment, that the peak plasma concentration of Ro-05-9963 is ~50% of that for equimolar injected doses of MIS. However, the Ro-05-9963 data extrapolate to a zero time value on the concentration axis which is twice that for MIS, which suggests the same apparent volume of distribution (Vd) for the two drugs. Estimates of Vd should, however, be treated with extreme caution when drugs are not administered by the i.v. route.

This demonstration of the shorter plasma half-life of Ro-05-9963, however, does not allow us to decide between the alternative hypotheses posed above for the lower tumour concentrations of Ro-05-9963. To do this it was necessary to prolong the plasma half-lives of the two drugs, and then observe the effect on the tumour concentrations. This was done by preventing all urinary excretion for a few hours by surgically tying off both kidneys before drug injection.

*Prolongation of drug half-lives*

Mice with bilateral nephrectomies, or with both kidneys ligated, will survive ~48 h. For at least the first 8 h after the mice recover from the anaesthetic they do not appear grossly different in behaviour or activity from control mice or from mice given a sham operation. Thus, to test the hypothesis that the lower tumour concentrations of Ro-05-9963 might be due to more rapid clearance of drug from the plasma, normal mice and mice with both kidneys ligated ("nephrectomized") were injected within 3 h with equimolar doses of MIS or Ro-05-9963.

Table II shows plasma and tumour levels of MIS and Ro-05-9963 at 38 min and 6 h after injection. From these data the following conclusions can be drawn:

(i) Plasma and tumour levels of Ro-05-9963 38 min after injection of normal mice are less than 50% of those of MIS, despite equimolar injected doses (confirming the data in Table I).

TABLE II.—*Effect of bilateral kidney ligation (BKL) on plasma and tumour concentrations (mM)* of misonidazole and Ro-05-9963*

| | | 38 min after injection | | | 6 h after injection | | |
|---|---|---|---|---|---|---|---|
| Drug and dose | BKL | Plasma | Tumour | Tumour/Plasma | Plasma | Tumour | Tumour/Plasma |
| MIS | No | 5·3 | 3·4 | 0·64 | 0·41 | 0·48 | 1·17 |
| 1000 mg/kg | | 4·6 | 3·5 | 0·76 | 0·64 | 0·55 | 0·86 |
| (5 mmol/kg) | | 4·1 | 0·5 | 0·12 | 1·01 | 0·61 | 0·60 |
| | | 4·7 | 6·4 | 1·36 | 0·26 | 0·24 | 0·92 |
| | | 4·2 | 3·4 | 0·81 | 0·50 | 0·27 | 0·54 |
| | | 4·9 | 3·6 | 0·73 | 0·41 | 0·21 | 0·51 |
| | Mean | 4·6 | 3·5 | 0·74 | 0·54 | 0·39 | 0·77 |
| | s.e. | 0·2 | 0·8 | 0·16 | 0·11 | 0·07 | 0·11 |
| | Yes | 5·7 | 2·0 | 0·35 | 5·2 | 4·3 | 0·83 |
| | | 3·4 | 3·3 | 0·97 | 5·2 | 3·4 | 0·65 |
| | | 7·0 | 4·2 | 0·60 | 5·0 | 2·6 | 0·52 |
| | | 5·5 | 3·2 | 0·58 | 4·9 | 2·8 | 0·57 |
| | | 5·3 | 3·6 | 0·68 | 5·1 | 2·7 | 0·53 |
| | | 5·9 | 3·2 | 0·54 | 5·2 | 3·2 | 0·62 |
| | Mean | 5·5 | 3·3 | 0·62 | 5·1 | 3·2 | 0·62 |
| | s.e. | 0·5 | 0·3 | 0·08 | 0·1 | 0·3 | 0·05 |
| Ro-05-9963 | No | 0·42 | 0·24 | 0·57 | <0·05 | <0·05 | — |
| 935 mg/kg | | 0·51 | 0·25 | 0·49 | <0·05 | <0·05 | — |
| (5 mmol/kg) | | 1·93 | 2·35 | 1·22 | <0·05 | <0·05 | — |
| | | 2·16 | 1·30 | 0·60 | <0·05 | <0·05 | — |
| | | 2·36 | 1·58 | 0·67 | | | |
| | | 1·27 | 0·90 | 0·71 | | | |
| | Mean | 1·4 | 1·1 | 0·71 | <0·05 | <0·05 | — |
| | s.e. | 0·3 | 0·3 | 0·11 | | | |
| | Yes | 4·7 | 4·0 | 0·85 | 5·9 | 2·1 | 0·36 |
| | | 4·7 | 3·1 | 0·66 | 4·1 | 3·7 | 0·90 |
| | | 5·4 | 4·3 | 0·80 | 4·6 | 2·3 | 0·50 |
| | | 5·1 | 2·4 | 0·47 | 4·7 | 2·9 | 0·62 |
| | | 5·1 | 2·8 | 0·55 | | | |
| | | 4·9 | 1·6 | 0·33 | | | |
| | Mean | 5·0 | 3·0 | 0·61 | 4·8 | 2·8 | 0·60 |
| | s.e. | 0·1 | 0·4 | 0·08 | 0·4 | 0·4 | 0·11 |

* Concentrations determined by spectrophotometric assay.

(ii) In kidney-ligated mice there is little or no decrease in the total 2-nitroimidazole concentration in plasma and tumour up to 6 h after injection. This indicates that the loss of 2-nitroimidazoles from the plasma is more dependent on urinary excretion than on any other routes of elimination, including extra-renal metabolism. We cannot, of course, exclude the kidney as a site of drug metabolism.

(iii) Bilateral kidney ligation increases the concentration of Ro-05-9963 in the tumour and plasma, at both 38 min and 6 h, to the same level as MIS. On the other hand, there is little or no change in MIS levels in the kidney-ligated mice at 38 min.

(iv) Bilateral kidney ligation, despite greatly prolonging the half-lives of the two drugs, does not change the tumour/plasma ratios, and as in unligated mice, the tumour/plasma ratio is the same for MIS and Ro-05-9963 (60–70%).

In order to characterize more fully the effect of bilateral kidney ligation on the pharmacokinetics of MIS, we injected a dose of 1000 mg/kg (5 mmol/kg) i.p. into normal, kidney-ligated, or sham-operated mice. Blood samples were collected from the mice for up to 12 h after injection and

FIG. 2.—(A) The whole blood concentration (±s.e.) of MIS after an i.p. injection of 5 mmol/kg into (Cambridge) female BALB/c mice which were either untreated (○), sham-operated (□), or had both kidneys ligated (△) before injection. The inverted symbol with (+G) above the point at 12½ h shows the addition of MIS glucuronide to the MIS level. (B) The concentration of Ro–05–9963 (±s.e.) in whole blood of the same mice as in (A). The mice were either untreated (●), sham-operated (■), or had bilateral kidney ligation (▲) before MIS injection. The inverted symbol (▼) shows the addition of Ro–05–9963 glucuronide. Both sets of data were obtained using HPLC on successive blood samples from 5 mice per treatment group.

FIG. 3.—The concentration of MIS+Ro-05-9963 (±s.e.) in the blood of mice as a function of time after a single injection of 5 mmol/kg (1000 mg/kg) of MIS. The mice were either untreated (○), sham-operated (□) or had both kidneys ligated (△) before injection. These data sum the corresponding data in Fig. 2.

assayed for MIS and its O-demethylated metabolite Ro-05-9963, using HPLC. In addition, the samples taken at ~12 h were treated with β-glucuronidase, as described in the Materials and Methods section, to determine the levels of MIS and Ro-05-9963 glucuronides.

TABLE III.—*Half-lives (in h with 95% confidence limits)* for total non-conjugated 2-nitroimidazole (MIS+Ro-05-9963)† in normal and kidney-ligated mice*

| Control | Sham-operated | Kidney-ligated |
|---|---|---|
| 3·5 (3·2–3·9) | 2·3 (2·1–2·5) | 9·6 (8·2–11·5) |
| 2·6 (2·5–2·7) | 2·9 (2·7–3·2) | 8·8 (6·6–13·0) |
| 2·6 (2·3–3·0) | 3·6 (3·3–4·0) | 11·9 (10·8–13·3) |
| 2·0 (1·8–2·2) | 2·6 (2·3–3·0) | 10·3 (9·1–11·9) |
| 2·1 (1·9–2·3) | — | 11·3 (10·0–13·0) |
| 2·5 (1·9–3·3) | 2·8 (2·1–3·8) | 10·3 (8·8–12·0) |
| 2·6 (2·2–3·1) | | |

*Determined using least-squares regression on the data 1–9 h after injection.

†Concentrations measured by HPLC.

Figs. 2 and 3 and Table III show the results. There was a clear difference between the half-lives of MIS (Fig. 2A) and of MIS +Ro-05-9963 (Fig. 3) in the kidney-ligated mice and in control or sham-operated mice. As can be seen from Table III, there was no significant difference between the half-lives in the control and sham-operated mice (the higher drug concentrations at each sampling time in the sham-operated mice over those in the controls (Figs. 2 and 3) are not significant, but, if real, could be the result of the anaesthetic or operation trauma).

The levels of the metabolite Ro-05-9963 build up considerably in the "nephrectomized" mice, to reach a steady-state concentration of about 0·8 mM, with no indication of any decrease at 12·5 h (Fig. 2B). In contrast, steady-state plasma concentrations in normal and sham-operated mice were about 0·27 mM for up to 6 h, before declining rapidly. However, the relative proportions of Ro-05-9963 to total non-conjugated 2-nitroimidazoles (MIS+Ro-05-9963) were not significantly different at any time in control and nephrectomized mice, rising from 5% at 38 min after injection in both groups to 24% in the normal and 22% in the nephrectomized mice by 6·5 h.

As well as detecting the Ro-05-9963 metabolite, we also observed the glucuronide conjugates of both MIS and Ro-05-9963 in the plasma of nephrectomized mice (Figs. 2 and 3) at levels which were ~30% of total 2-nitroimidazoles. These conjugates were not found in the plasma of normal and sham-operated mice.

As a further test of the comparability of the kidney-ligated and normal mice, we measured rectal temperatures at hourly intervals after MIS injection. During the first 5 h after injection all 3 groups had an identical temperature drop (5·0°C) due to the MIS injection. However, by 9 h after injection the rectal temperature of the controls had risen to within 3°C of uninjected mice, whereas the kidney-ligated mice had fallen still further to 7°C below the uninjected level.

*The effect of prolonged half-lives on radiosensitization*

In order to determine whether bilateral kidney ligation would overcome the lower drug levels of Ro-05-9963 in the hypoxic, radioresistant tumour cells, a radiation experiment was made to compare the radiosensitization produced as a function of time after an injection of 2·5 mmol/kg of Ro-05-9963 or MIS into normal and bilaterally kidney-ligated mice. The results are shown in Table IV. As predicted from the tumour concentrations, the extent of radiosensitization was less for Ro-05-9963 than for MIS at equimolar injected doses in the untreated mice, but was the same as (or slightly greater than) that for MIS when the blood flow to both kidneys was occluded before injection of Ro-05-9963.

TABLE IV.—*Effect of kidney ligation on radiosensitization of hypoxic tumour cells by Ro*-05-9963

| Drug and dose | Kidney ligation | Surviving fraction after 1750 rad[1] | Survival ratio[2] |
|---|---|---|---|
| Saline | No | $2·5 \times 10^{-2}$ | — |
| | Yes | $3·7 \times 10^{-2}$ | — |
| MIS 2·5 mmol/kg | No | $1·91 \times 10^{-3}$ (1·61–2·26) | 0·062 |
| Ro-05-9963 2·5 mmol/kg | No | $3·15 \times 10^{-3}$ (2·34–4·23) | 0·102 } $P = 0·05$ |
| | Yes | $1·47 \times 10^{-3}$ (1·14–1·89) | 0·047 } |

[1]Mean of values at 15, 30, 45 and 60 min after drug injection (with s.e. limits).

[2]Ratio of $\frac{\text{Surviving fraction in drug-injected mice}}{\text{Surviving fraction in saline-injected mice}}$

DISCUSSION

These data show that after equimolar injected doses of Ro-05-9963 and MIS, the plasma and tumour concentrations of Ro-05-9963 are less than those of MIS by a factor of 2 or more. In other experiments it was shown that this lower tumour concentration is responsible for the lower radiosensitization produced by Ro-05-9963 (Brown *et al.*, in preparation). It was further shown that the lower tumour concentration of Ro-05-9963 was not due to inadequate absorption or distribution resulting from its lower lipophilicity, but was due to the short plasma half-life of the drug. When all urinary excretion of the drugs was prevented, and plasma levels remained constant for an hour or more, no differences between the tumour concentrations in the MIS and Ro-05-9963 injected mice were seen.

The significance of this lies in the fact that in man the relatively long plasma half-life of MIS almost certainly contributes to the drug's toxicity (Saunders *et al.*, 1978). Thus, a valid direction in drug design would be to decrease the half-life without losing the high plasma and tumour levels achievable with MIS. Since the half-life in man is ~12 h (Foster *et al.*, 1975; Dische *et al.*, 1977; Wiltshire *et al.*, 1978; Workman *et al.*, 1978*b*), it should be possible to reduce this by a factor of 4 or 5 without changing peak tumour levels. However, in testing such drugs in the mouse or rat, such a reduction in the plasma half-life could result (as it does with Ro-05-9963) in low plasma and tumour concentrations. This could lead to rejection of the drug if a radiation assay alone were used to screen the efficacy of new compounds. Thus, it is necessary to determine tumour levels of the various drugs in conjunction with radiation assays.

However, although necessary, the determination of tumour levels of new drugs as a function of injected dose will not be *sufficient* to extrapolate their usefulness from mice to man. A relatively low tumour concentration, with or without low plasma levels, could be due either to very rapid clearance from the blood (as with Ro-05-9963), or it could be due to inadequate penetration of the drug through the various lipoid membranes between the site of application and the tumour cells. In the former case it would not be expected in man, because of the relatively long half-life, but in the latter case a similar problem could be expected in man.

The technique of bilateral kidney ligation may help to resolve the problem. This procedure prolongs the plasma half-life of MIS in the mouse from ~2 h to 10 h, similar to that in man. It remains to be seen whether this will apply generally to other 2-nitroimidazole radiosensitizers, but it is promising that the conclusions reached using bilateral kidney ligation in comparing Ro-05-9963 and MIS (namely that the lower plasma concentrations of Ro-05-9963 than MIS found in the mouse will not apply in man) are borne out in the dog, which has a plasma half-life intermediate between mouse and man (White and Workman, in preparation). Of course, kidney ligation produces an increasingly abnormal physiological state which could influence drug pharmacokinetics. For example, in man uraemia due to impaired renal function can sometimes shorten and sometimes prolong the apparent half-life of drugs eliminated partly by metabolism (Reidenberg, 1974, 1977). Kidney ligation also precludes any metabolism of the drugs by the kidney. However, since we found the ratio of Ro-05-9963 to unchanged MIS in the plasma of kidney-ligated mice to be the same as that in control mice, this possibility is probably not relevant in the present case.

The second question posed in the introductory section was to what extent does the plasma half-life affect the tumour/plasma ratio of drug concentrations? We conclude that, for the two drugs studied and at the injected doses (5 mmol/kg), a change in plasma half-life from ~30 min to 10 h does not influence the tumour/plasma ratio. Thus, in Tables I and II it can be seen that at all times after injection of either MIS or Ro-05-9963, in either normal or kidney-ligated mice, the ratio of nitroimidazole in the tumour to that in plasma is 60–70%. This conclusion is contrary both to the generally held view (*e.g.* Adams, 1977; Dische *et al.*, 1977; Stratford & Adams, 1978; Denekamp & Fowler, 1978) and to the inference of McNally *et al.* (1978) that the longer half-life of MIS in man than in mice would result in a higher human tumour/plasma ratio. Their data, however, do not support this suggestion; they found in different mouse tumours concentrations 18–72% of that in blood, compared to a range of 42–107% of plasma levels in human tumours (Gray *et al.*, 1976; Dische *et al.*, 1977; Urtasun *et al.*, 1977). Further, more recent clinical studies, using HPLC analysis, have shown that after the administration of MIS the concentrations of the parent drug and the metabolite Ro-05-9963 were normally in the range 50–70% of the corresponding plasma concentrations (Wiltshire *et al.*, 1978; Workman *et al.*, 1978*b*). Thus there seems little evidence that the tumour/plasma ratio in man is generally higher than in the mouse; in both species the ratio may be a function of the individual tumour rather than of the drug's plasma half-life.

The present studies have clearly shown that tumour levels of MIS and Ro-05-9963 maintain a constant ratio to plasma concentration, but are ~30–40% lower. It is therefore of interest to ask why the concentrations in tumour and plasma are not equal. There appear to be two possibilities:

(i) The partitioning of the 2-nitroimidazoles from plasma to tumour is not equal.

(ii) The partitioning is equal, but the nitroimidazoles are metabolized in the tumour.

Data are available in support of both hypotheses. In support of the metabolism hypothesis, Varghese *et al.* (1976) and Flockhart *et al.* (1978*a*) have provided evidence that reduction of the nitro group in mouse tumours *in vivo* depends upon the particular tumour type.

In support of the partitioning hypothesis, the data of Flockhart *et al.* (1978*c*) have shown that the uptake of (2-$^{14}$C) MIS by the MT mouse tumour and some normal mouse tissues was less than 100% of the plasma concentration, particularly at less than 6 h after injection. In contrast, concentrations in liver were considerably higher than in plasma. Also pertinent is the recent finding that the saliva/plasma ratio for MIS in man was less than unity (0·87±0·05) (Workman *et al.*, 1978*b*).

The extent of tumour necrosis may have an important influence on the uptake and metabolism of 2-nitroimidazoles. In man the reported concentration of MIS in necrotic fluid varies widely (Flockhart *et al.*, 1978*a*). Rauth *et al.* (1978) have shown that the concentration of MIS in the necrotic fluid of the KHT mouse tumour can be considerably lower than that in the whole tumour. The Stanford EMT6 tumour used in the present study contained a large necrotic centre occupying roughly 30% of the tumour volume (Brown *et al.*, 1978). The Cambridge EMT6 tumour is rather less necrotic, but necrosis increases with tumour size, and it is relevant that MIS concentrations are considerably lower in 300mm$^3$ tumours than in 100mm$^3$ ones (Workman *et al.*, in preparation).

The authors are grateful to Professor N. M. Bleehen for helpful discussions and to Ms R. Goddard, Mrs J. Donaldson and Mr E. Parker for their skilled technical contributions. They would also like to thank Roche Products Limited, and in particular Dr C. E. Smithen, for the generous donation of the Ro-05-9963, and the U.S. National Cancer Institute for their continued supply of misonidazole. This investigation was funded by Research Grant No. CA-15201 and Research Contract No. CM-87207 from the National Cancer Institute, DHEW and by the MRC.

### REFERENCES

Adams, G. E. (1977) Hypoxic cell radiosensitizers for radiotherapy. In *Cancer: A Comprehensive Treatise.* Ed. F. Becker. New York: Plenum Press, Vol. 6, p. 181.

Adams, G. E., Flockhart, I. R., Smithen, C. E., Stratford, I. J., Wardman, P. & Watts, M. E. (1976) Electron-affinic sensitization. VII. A correlation between structures, one-electron reduction potentials and efficiencies of nitroimidazoles as hypoxic cell radiosensitizers. *Radiat. Res.*, **67**, 9.

Asquith, J. C., Watts, M. E., Patel, K., Smithen, C. E. & Adams, G. E. (1974) Electron affinic sensitization. V. Radiosensitization of hypoxic bacteria and mammalian cells *in vitro* by some nitroimidazoles and nitro pyrazoles. *Radiat. Res.*, **60**, 108.

BROWN, J. M. (1975) Selective radiosensitization of the hypoxic cells of mouse tumours with the nitroimidazoles metronidazole and Ro-7-0582. *Radiat. Res.*, **64**, 633.

BROWN, J. M., YU, N. Y., CORY, M. J., BICKNELL, R. B. & TAYLOR, D. L. (1978) *In vivo* evaluation of the radiosensitizing and cytotoxic properties of newly synthesized electron affinic drugs. *Br. J. Cancer*, **37**, (Suppl. III), 206.

CHAPMAN, J. D., REUVERS, A. P., BORSA, J., HENDERSON, J. H. & MIGLIORE, R. D. (1974) Nitroheterocyclic drugs as selective radiosensitizers of hypoxic mammalian cells. *Cancer Chemother. Rep.*, **58**, 559.

CHIN, J., VARGHESE, A. J. & RAUTH, A. M. (1978) 26th Ann. Meeting, Radiat. Res. Soc. Toronto. (Abstract Col-4.)

DENEKAMP, J., MICHAEL, B. D. & HARRIS, S. R. (1974) Hypoxic cell radiosensitizers: comparative tests of some electron-affinic compounds using epidermal cell survival *in vivo*. *Radiat. Res.*, **60**, 119.

DENEKAMP, J. & FOWLER, J. F. (1978) Radiosensitization of solid tumours by nitroimidazoles. *Int. J. Radiat. Oncol. Biol. Phys.*, **4**, 143.

DISCHE, S., SAUNDERS, M. I., LEE, M. E., ADAMS, G. E. & FLOCKHART, I. R. (1977). Clinical testing of the radiosensitizer Ro-07-0582. Experience with multiple doses. *Br. J. Cancer*, **35**, 567.

FLOCKHART, I. R., MALCOLM, S. L., MARTEN, T. R., PARKINS, C. S., RUANE, R. J. & TROUP, D. (1978*a*) Some aspects of the metabolism of misonidazole. *Br. J. Cancer*, **37**, (Suppl. III), 264.

FLOCKHART, I. R., SHELDON, P. W., STRATFORD, I. J. & WATTS, M. E. (1978*b*) A metabolite of the 2-nitroimidazole misonidazole with radiosensitizing properties. *Int. J. Radiat. Biol.*, **34**, 91.

FLOCKHART, I. R., LARGE, P., TROUP, D., MALCOLM, S. L. & MARTEN, T. R. (1978*c*) Pharmacokinetic and metabolic studies of the hypoxic cell radiosensitizer misonidazole. *Xenobiotica*, **8**, 97.

FOSTER, J. L., FLOCKHART, I. R., DISCHE, S., GRAY, A., LENOX-SMITH, I. & SMITHEN, C. E. (1975) Serum concentration measurements in man of the radiosensitizer Ro-07-0582: some preliminary results. *Br. J. Cancer*, **31**, 679.

GRAY, A. J., DISCHE, S., ADAMS, G. E., FLOCKHART, I. R. & FOSTER, J. L. (1976) Clinical testing of the radiosensitizer Ro-07-0582. I. Dose tolerance, serum and tumour concentrations. *Clin. Radiol.*, **27**, 151.

MCNALLY, N. J., DENEKAMP, J., SHELDON, P. W., FLOCKHART, I. R. & STEWART, F. A. (1978) Radiosensitization by misonidazole (Ro-07-0582). The importance of timing and tumour concentration of sensitizer. *Radiat. Res.*, **73**, 568.

RAUTH, A. M., CHIN, J., MARCHOW, L. & PACIGA, J. (1978) Testing of hypoxic cell radiosensitizers *in vivo*. *Br. J. Cancer*, **37**, (Suppl. III), 202.

REIDENBERG, M. M. (1974) Effect of excretion changes on drug action and drug interactions, In *Drug Interactions* Ed. P. L. Morselli, S. Garattini and S. N. Cohen. New York: Raven. p. 241.

REIDENBERG, M. M. (1977) The effects of the kidney on drug actions and drug interactions, In *Drug Interactions* Ed. D. G. Grahame-Smith. London: MacMillan. p. 209.

SAUNDERS, M. I., DISCHE, S., ANDERSON, P. & FLOCKHART, I. R. (1978) The neurotoxicity of misonidazole and its relationship to dose, half-life and concentration in the serum. *Br. J. Cancer*, **37**, (Suppl. III), 268.

STRATFORD, I. J. & ADAMS, G. E. (1978) The toxicity of the radiosensitizer misonidazole towards hypoxic cells *in vitro*: a model for mouse and man. *Br. J. Radiol.*, **51**, 745.

THOMLINSON, R. H., DISCHE, S., GRAY, A. J. & ERRINGTON, L. M. (1976) Clinical testing of the radiosensitizer Ro-07-0582. III. Response of tumours. *Clin. Radiol.*, **27**, 167.

URTASUN, R. C., BAND, P., CHAPMAN, J. D., RABIN, H. R., WILSON, A. F. & FRYER, C. G. (1977) Clinical phase 1 study of the hypoxic cell radiosensitizer Ro 07-0582, a 2-nitroimidazole derivative. *Radiology*, **122**, 801.

URTASUN, R. C., CHAPMAN, J. D., FELDSTEIN, M. L. & 6 others (1978) Peripheral neuropathy related to misonidazole: Incidence and pathology. *Br. J. Cancer*, **37**, (Suppl. III), 271.

VARGHESE, A. J., GULYAS, S. & MOHINDRA, J. K. (1976) Hypoxia-dependent reduction of 1-(2-nitro-1-imidazolyl)-3-methoxy-2-propanol by Chinese hamster ovary cells and KHT tumour cells *in vitro* and *in vivo*. *Cancer Res.*, **36**, 3701.

WARDMAN, P. (1977) The use of nitroaromatic compounds as hypoxic cell radiosensitizers. In *Current Topics in Radiation Research Quarterly.* Eds. M. Ebert and A. Howard. Amsterdam: North-Holland, p. 347.

WILTSHIRE, C. R., WORKMAN, P., WATSON, J. V. & BLEEHEN, N. M. (1978) Clinical studies with misonidazole. *Br. J. Cancer*, **37**, (Suppl. III), 286.

WORKMAN, P., LITTLE, C. J., MARTEN, T. R. & 4 others (1978*a*) Estimation of the hypoxic cell-sensitizer misonidazole and its O-demethylated metabolite in biological materials by reversed-phase high-performance liquid chromotography. *J. Chromatogr.*, **145**, 507.

WORKMAN, P., WILTSHIRE, C. R., PLOWMAN, P. N. & BLEEHEN, N. M. (1978*b*) Monitoring salivary misonidazole in man: a possible alternative to plasma monitoring. *Br. J. Cancer*, **38**, 709.


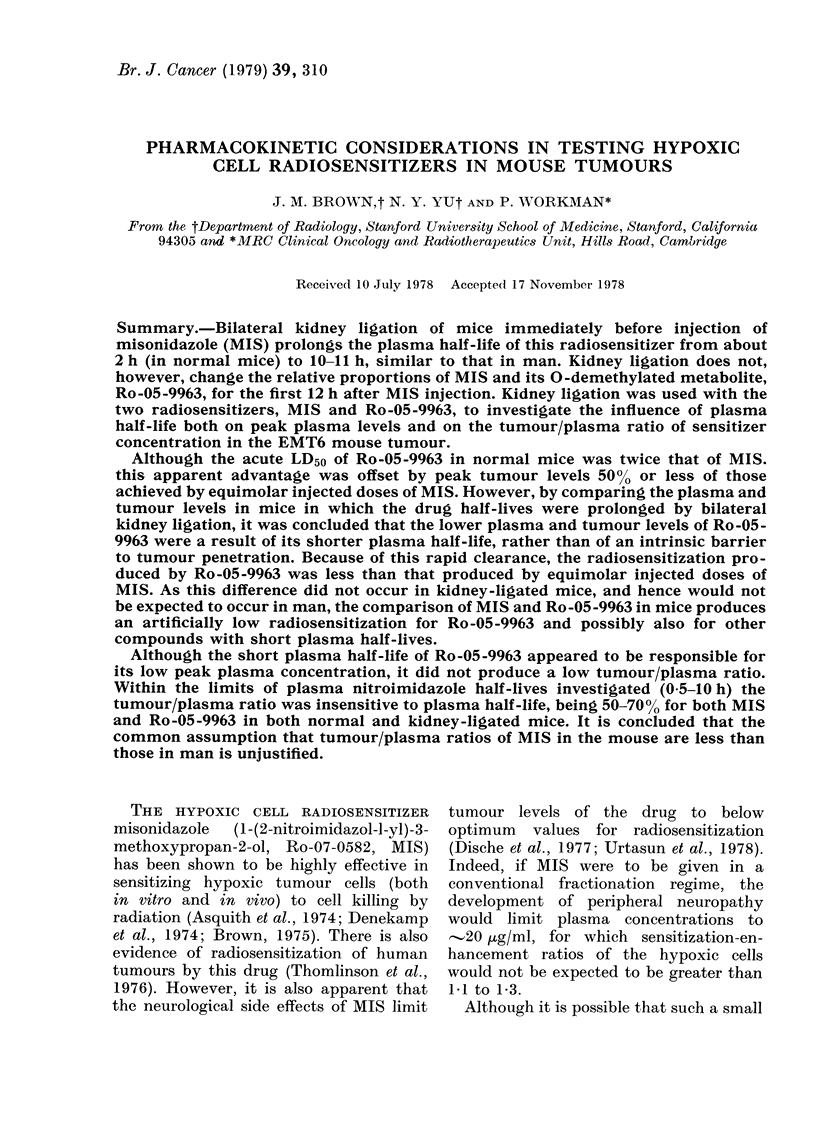

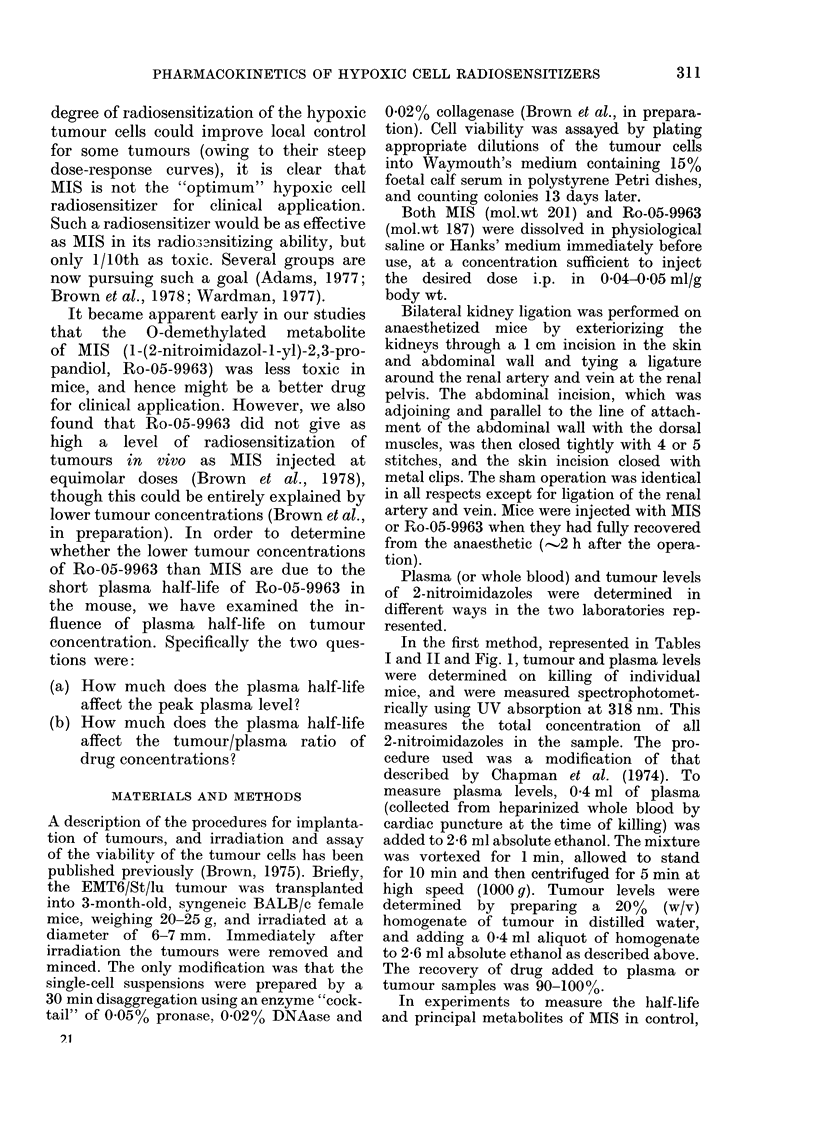

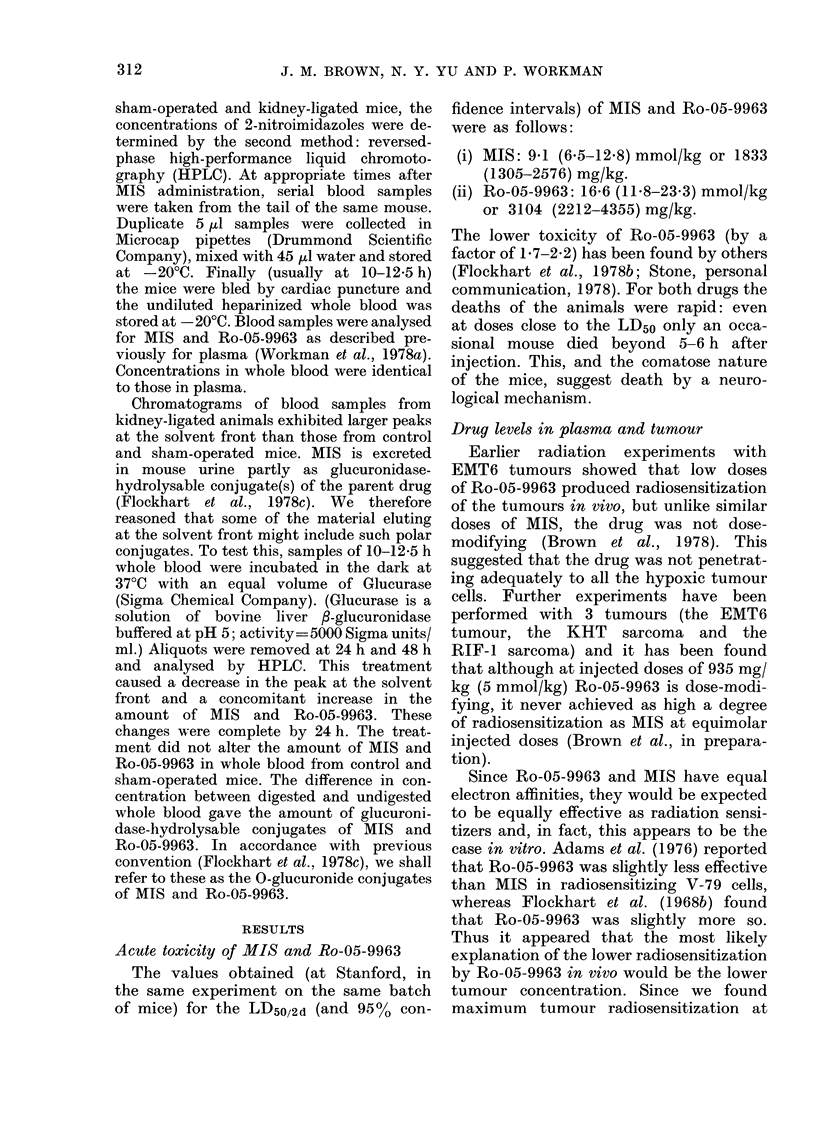

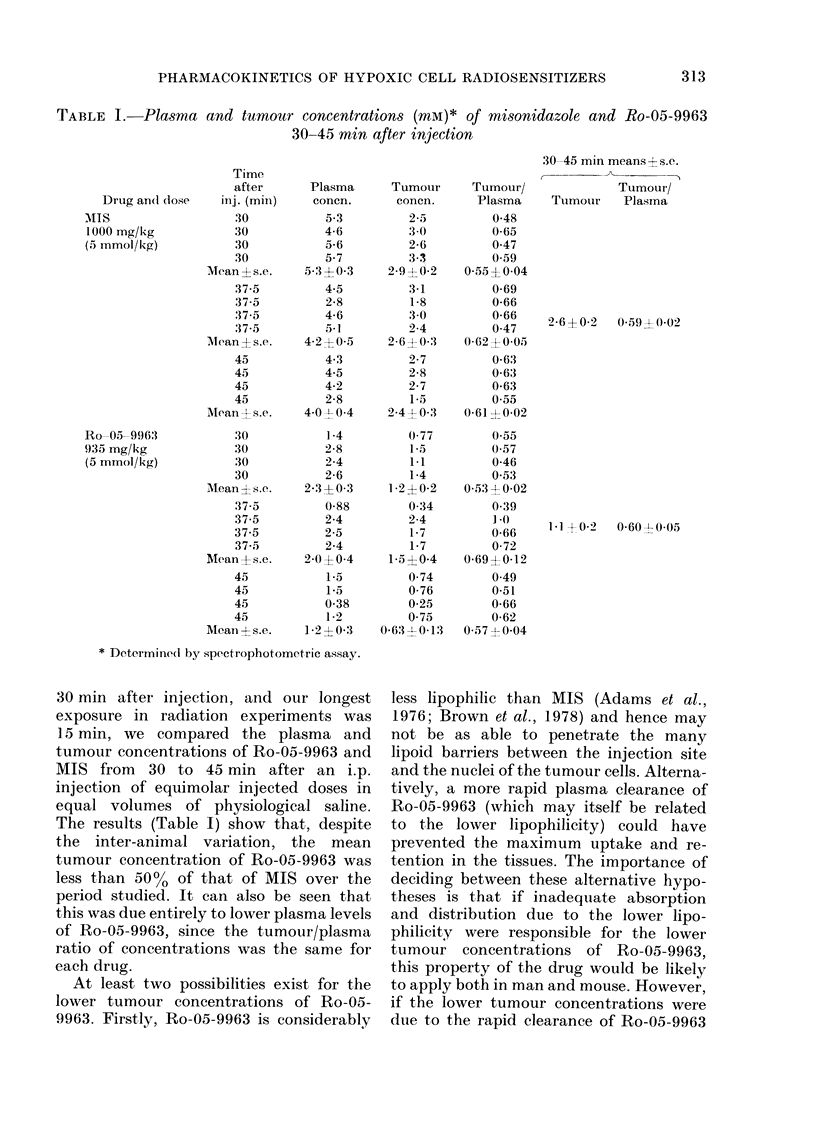

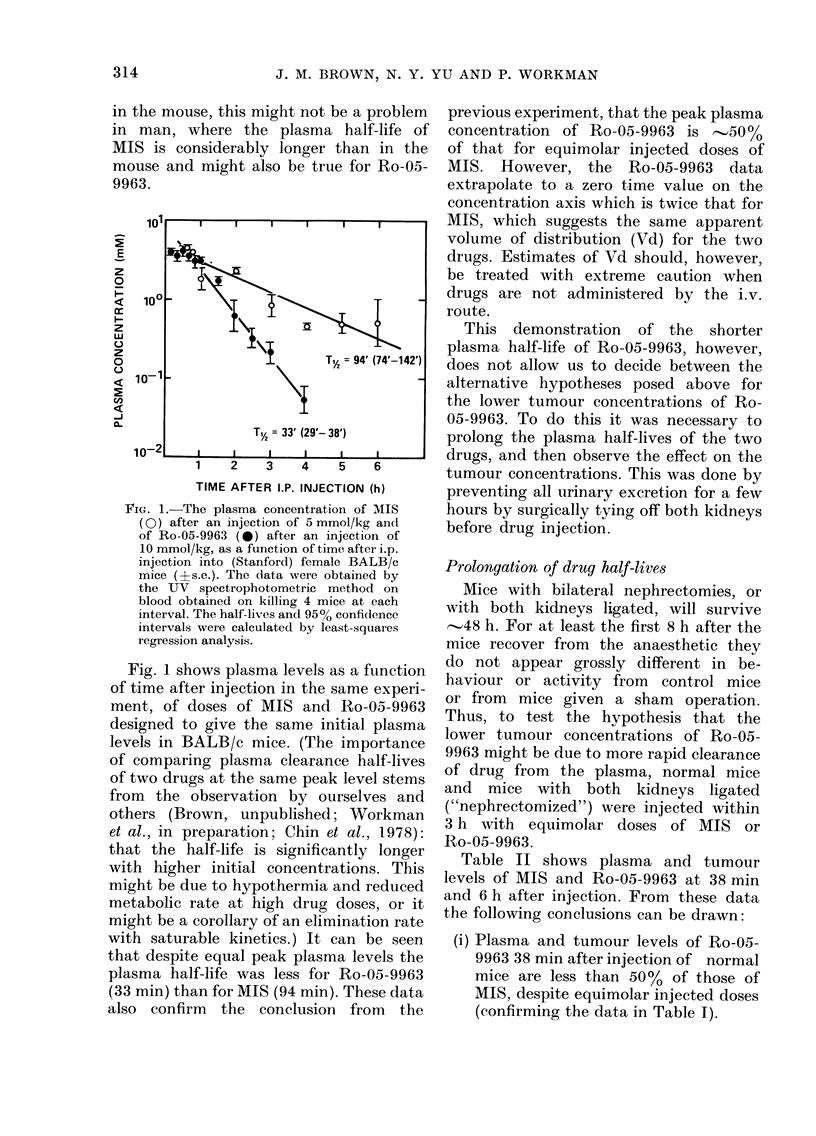

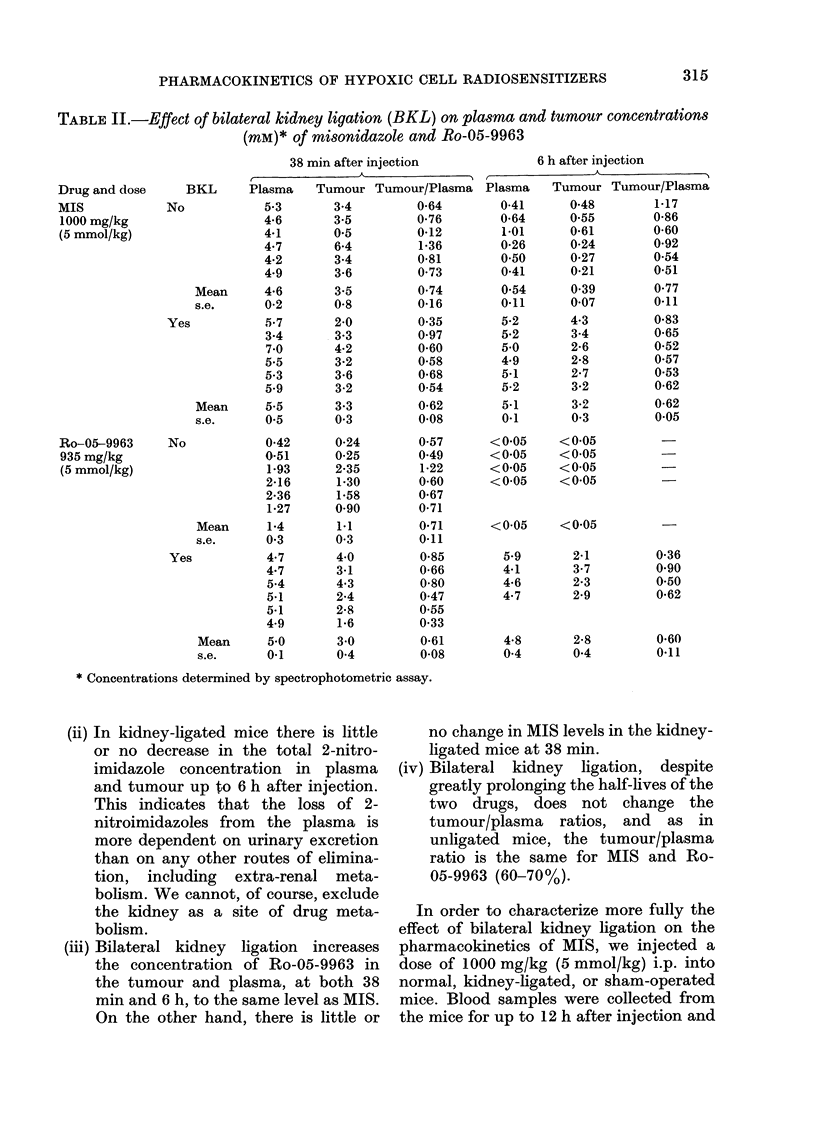

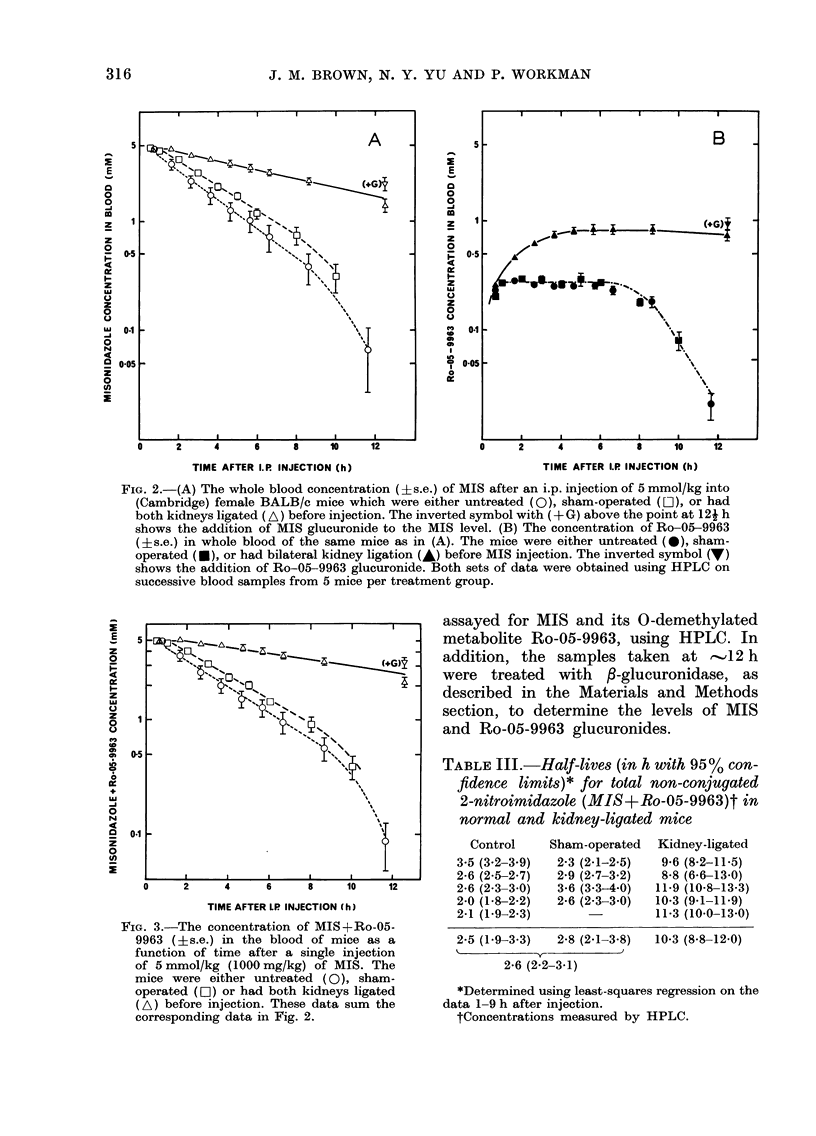

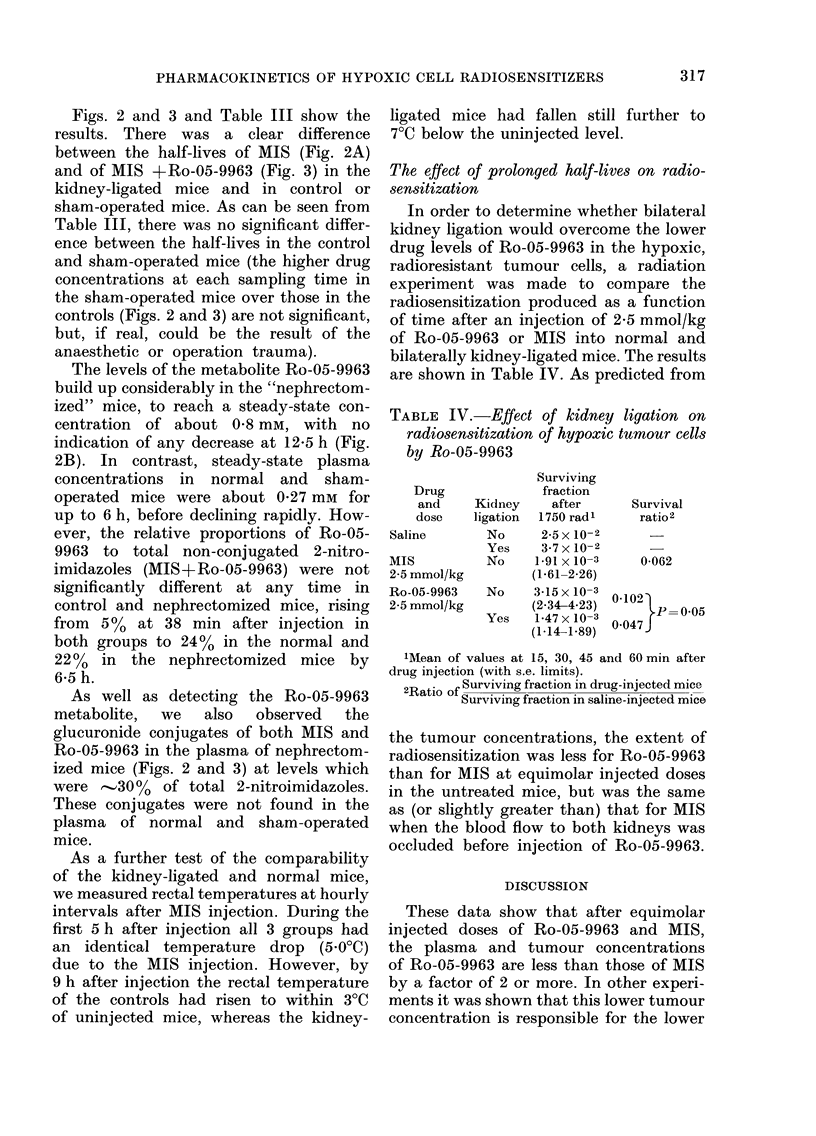

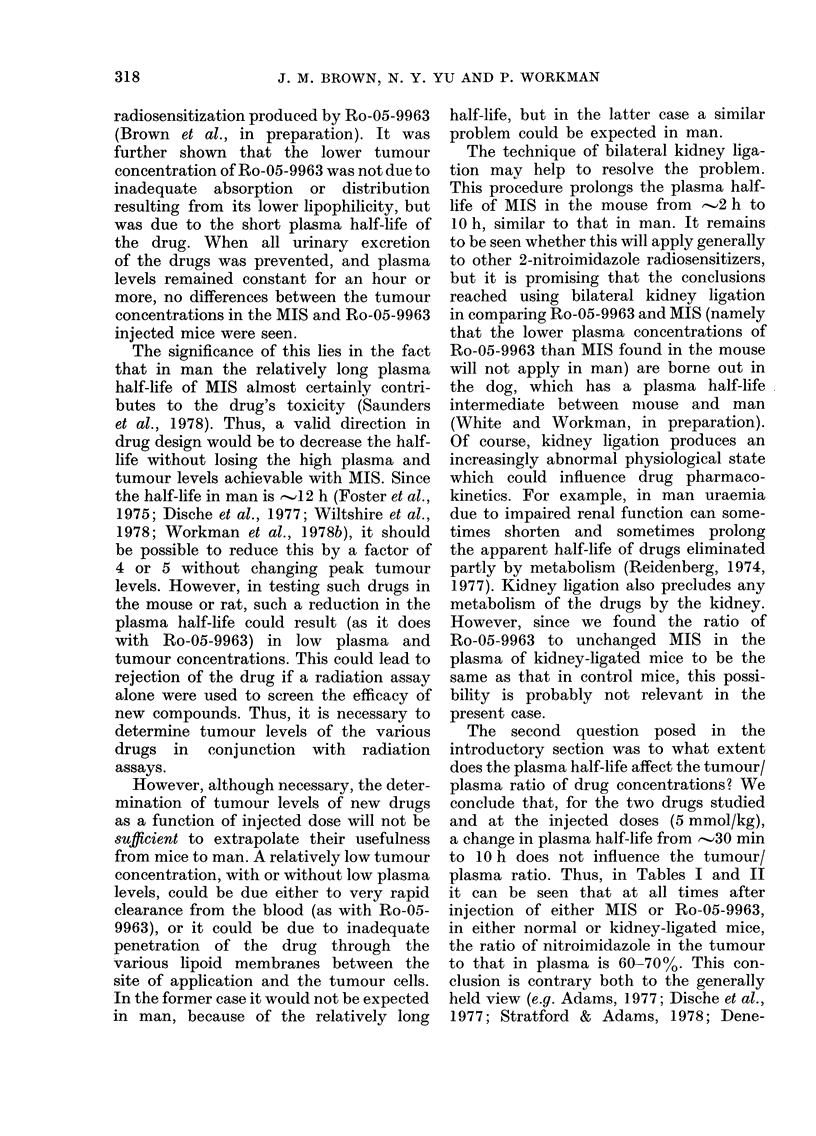

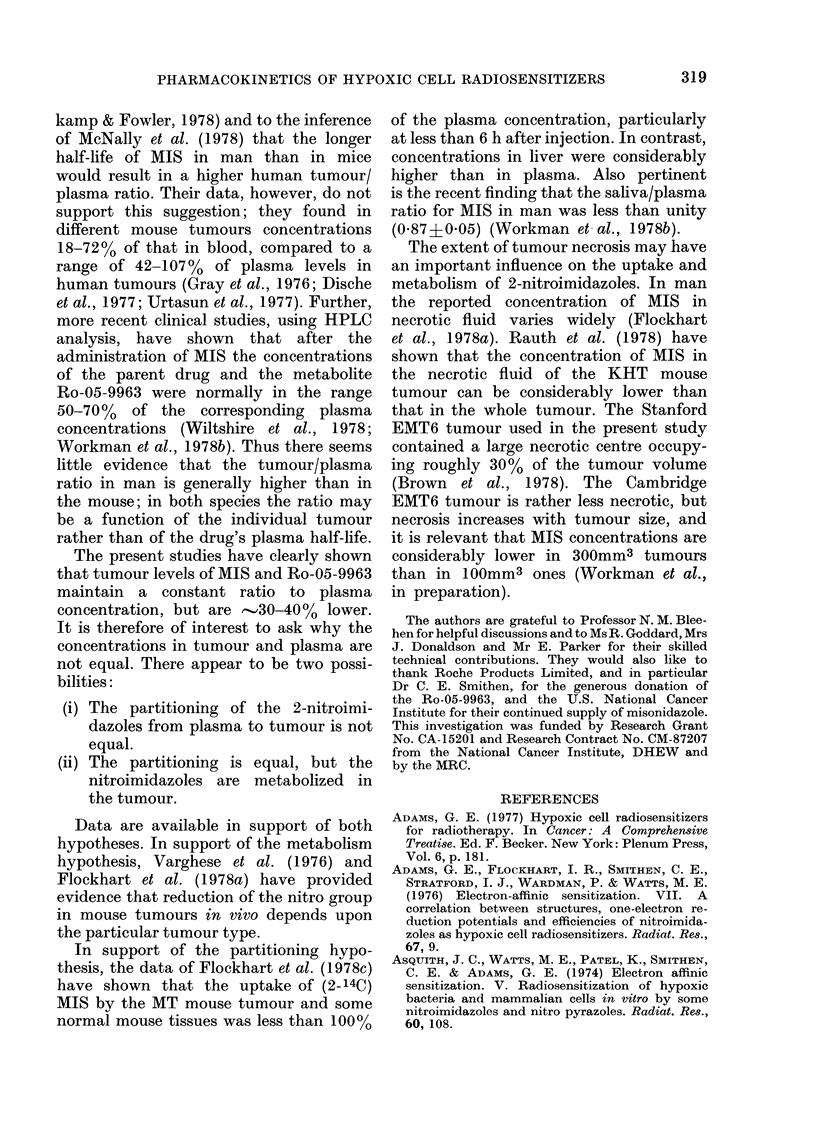

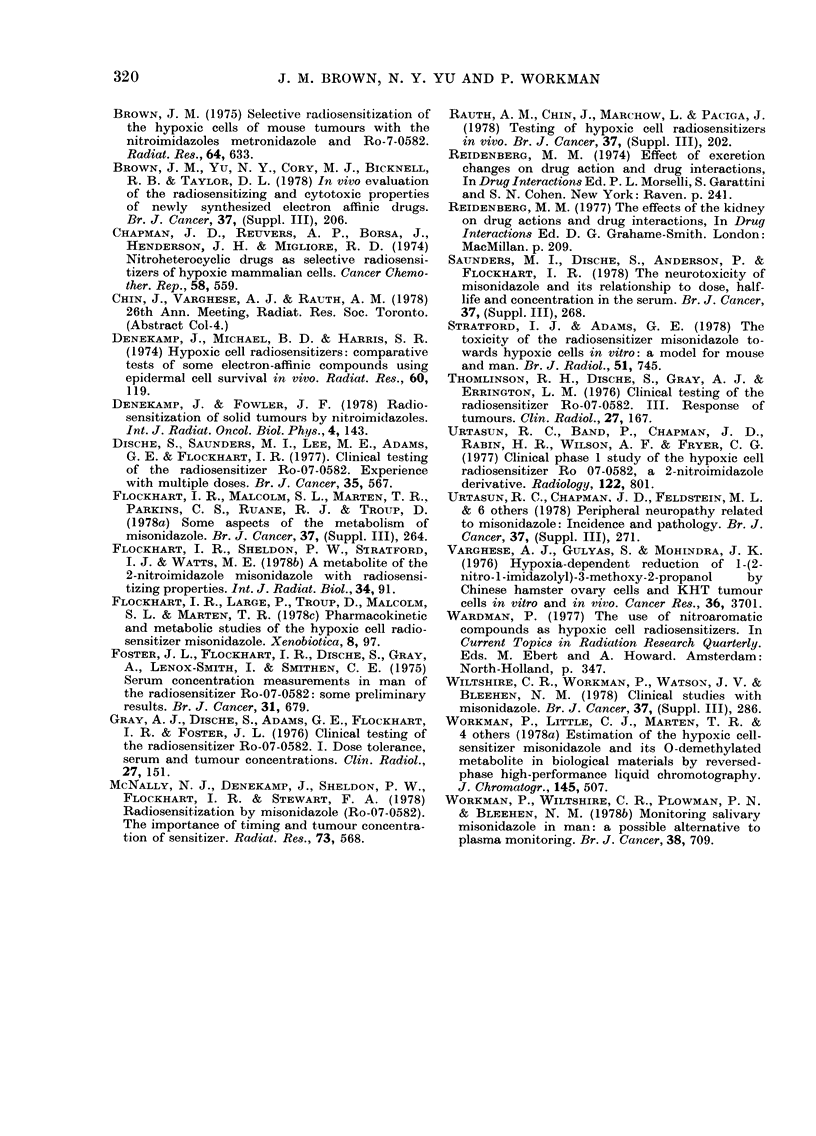


## References

[OCR_01461] Adams G. E., Flockhart I. R., Smithen C. E., Stratford I. J., Wardman P., Watts M. E. (1976). Electron-affinic sensitization. VII. A correlation between structures, one-electron reduction potentials, and efficiencies of nitroimidazoles as hypoxic cell radiosensitizers.. Radiat Res.

[OCR_01470] Asquith J. C., Watts M. E., Patel K., Smithen C. E., Adams G. E. (1974). Electron affinic sensitization. V. Radiosensitization of hypoxic bacteria and mammalian cells in vitro by some nitroimidazoles and nitropyrazoles.. Radiat Res.

[OCR_01480] Brown J. M. (1975). Selective radiosensitization of the hypoxic cells of mouse tumors with the nitroimidazoles metronidazole and Ro 7-0582.. Radiat Res.

[OCR_01486] Brown J. M., Yu N. Y., Cory M. J., Bicknell R. B., Taylor D. L. (1978). In vivo evaluation of the radiosensitizing and cytotoxic properties of newly synthesized electron-affinic drugs.. Br J Cancer Suppl.

[OCR_01493] Chapman J. D., Reuvers A. P., Borsa J., Henderson J. S., Migliore R. D. (1974). Nitroheterocyclic drugs as selective radiosensitizers of hypoxic mammalian cells.. Cancer Chemother Rep.

[OCR_01512] Denekamp J., Fowler J. F. (1978). Radiosensitization of solid tumors by nitroimidazoles.. Int J Radiat Oncol Biol Phys.

[OCR_01505] Denekamp J., Michael B. D., Harris S. R. (1974). Hypoxic cell radiosensitizers: comparative tests of some electron affinic compounds using epidermal cell survival in vivo.. Radiat Res.

[OCR_01517] Dische S., Saunders M. I., Lee M. E., Adams G. E., Flockhart I. R. (1977). Clinical testing of the radiosensitizer Ro 07-0582: experience with multiple doses.. Br J Cancer.

[OCR_01535] Flockhart I. R., Large P., Troup D., Malcolm S. L., Marten T. R. (1978). Pharmacokinetic and metabolic studies of the hypoxic cell radiosensitizer misonidazole.. Xenobiotica.

[OCR_01529] Flockhart I. R., Sheldon P. W., Stratford I. J., Watts M. E. (1978). A metabolite of the 2-nitroimidazole misonidazole with radiosensitizing properties.. Int J Radiat Biol Relat Stud Phys Chem Med.

[OCR_01541] Foster J. L., Flockhart I. R., Dische S., Gray A., Lenox-Smith I., Smithen C. E. (1975). Serum concentration measurements in man of the radiosensitizer Ro-07-0582: some preliminary results.. Br J Cancer.

[OCR_01548] Gray A. J., Dische S., Adams G. E., Flockhart I. R., Foster J. L. (1976). Clinical testing of the radiosensitiser Ro-07-0582. I. Dose tolerance, serum and tumour concentrations.. Clin Radiol.

[OCR_01555] McNally N. J., Denekamp J., Sheldon P., Flockhart I. R., Stewart F. A. (1978). Radiosensitization by misonidazole (Ro 07-0582). The importance of timing and tumor concentration of sensitizer.. Radiat Res.

[OCR_01564] Rauth A. M., Chin J., Marchow L., Paciga J. (1978). Testing of hypoxic cell radiosensitizers in vivo.. Br J Cancer Suppl.

[OCR_01579] Saunders M. E., Dische S., Anderson P., Flockhart I. R. (1978). The neurotoxicity of misonidazole and its relationship to dose, half-life and concentration in the serum.. Br J Cancer Suppl.

[OCR_01586] Stratford I. J., Adams G. E. (1978). The toxicity of the radiosensitizer misonidazole towards hypoxic cells in vitro: a model for mouse and man.. Br J Radiol.

[OCR_01592] Thomlinson R. H., Dische S., Gray A. J., Errington L. M. (1976). Clinical testing of the radiosensitiser Ro-07-0582. III. Response of tumours.. Clin Radiol.

[OCR_01598] Urtasun R. C., Band P., Chapman J. D., Rabin H. R., Wilson A. F., Fryer C. G. (1977). Clinical phase I study of the hypoxic cell radiosensitizer RO-07-0582, a 2-nitroimidazole derivative.. Radiology.

[OCR_01605] Urtasun R. C., Chapman J. D., Feldstein M. L., Band R. P., Rabin H. R., Wilson A. F., Marynowski B., Starreveld E., Shnitka T. (1978). Peripheral neuropathy related to misonidazole: incidence and pathology.. Br J Cancer Suppl.

[OCR_01624] Wiltshire C. R., Workman P., Watson J. V., Bleehen N. M. (1978). Clinical studies with misonidazole.. Br J Cancer Suppl.

[OCR_01628] Workman P., Little C. J., Marten T. R., Dale A. D., Ruane R. J., Flockhart I. R., Bleehen N. M. (1978). Estimation of the hypoxic cell-sensitiser misonidazole and its O-demethylated metabolite in biological materials by reversed-phase high-performance liquid chromatography.. J Chromatogr.

[OCR_01636] Workman P., Wiltshire C. R., Plowman P. N., Bleehen N. M. (1978). Monitoring salivary misonidazole in man: a possible alternative to plasma monitoring.. Br J Cancer.

